# AMP-36 exhibits potent therapeutic efficacy against MRSA pneumonia through membrane-target mechanism

**DOI:** 10.1038/s41598-026-44156-6

**Published:** 2026-03-17

**Authors:** Yanxiao Han, Yuli Wang, Lin Cheng, Chenxi Sun, Jialin Song, Xunqi Zhang, Xuhua Zhang, Yang Jiang, Xiaoyan Li, Dexiao Kong, Chengyun Zheng

**Affiliations:** 1 Department of Hematology, The Second Qilu Hospital of Shandong University, Jinan, Shandong China; 2 Institute of Biotherapy for Hematological Malignancy, Shandong University, Jinan, Shandong China; 3 Shandong University-Karolinska Institute Collaboration Laboratory for Stem Cell Research, Jinan, Shandong China; 4 Department of Clinical Laboratory, The Second Qilu Hospital of Shandong University, Jinan, Shandong China; 5 Emergency Department, Shengli Oilfield Central Hospital, Dongying, Shandong China; 6 Shenzhen Weigao Shengji Medical Technology Co., Ltd, Shenzhen, China

**Keywords:** Antimicrobial peptide, Drug-resistant bacteria, MRSA, Pneumonia, Mechanism, Biotechnology, Drug discovery, Microbiology

## Abstract

The rapid emergence of multidrug-resistant (MDR) bacteria poses a critical challenge in hospital-acquired infections, particularly methicillin-resistant *Staphylococcus aureus* (MRSA) pneumonia. Antimicrobial peptides (AMPs) are promising therapeutic candidates due to their broad-spectrum antibacterial activity. Here, we report AMP-36, a 36-amino acid antimicrobial peptide rationally designed and synthesized from SAAP-148, exhibits potent antibacterial activity. AMP-36 displayed low micromolar minimum inhibitory concentrations and rapid bactericidal activity in vitro, achieving near-complete bacterial killing within 8 h. In the murine pneumonia model, AMP-36 significantly reduced bacterial burden in bronchoalveolar lavage fluid (BALF) and markedly alleviated lung inflammation. Scanning electron microscopy (SEM) revealed pronounced disruption of MRSA cell membranes following AMP-36 treatment, indicating membrane damage as the primary antibacterial mechanism. Transcriptomic analysis further demonstrated its broad transcriptional alterations. Collectively, these findings highlight AMP-36 as a promising therapeutic candidate for MRSA pneumonia and provide mechanistic insights into its antimicrobial action.

**Supplementary Information:**

The online version contains supplementary material available at 10.1038/s41598-026-44156-6.

## Introduction

Hospital-acquired pneumonia (HAP) is among the most common healthcare-associated infections, particularly affecting patients in intensive care units (ICUs), where it is associated with high morbidity, mortality, and treatment costs^1^. Beyond the ICU, non-ICU HAP is also a major cause of hospital-associated pneumonia, especially in medical wards and among elderly patients with severe comorbidities^2^, particularly the patients with hematological malignancies are vulnerable to HAP. In recent years, the global burden of multidrug-resistant (MDR) bacteria has risen sharply, complicating infection control and posing a serious threat to public health^3^. Among MDR pathogens, methicillin-resistant *Staphylococcus aureus* (MRSA) is of special concern, as it has been implicated in hospital outbreaks worldwide^4^. The emergence and persistence of MRSA significantly exacerbate the risk of acute pneumonia due to its impact on both morbidity and mortality^5^. Although vancomycin and linezolid remain the mainstays of therapy, their effectiveness is increasingly undermined by the emergence of vancomycin intermediate-resistant *S. aureus* (VISA) and vancomycin-resistant *S. aureus* (VRSA) causes their efficacy increasingly compromised^6^. Consequently, there is an urgent need for novel antimicrobial agents.

Antimicrobial peptides (AMPs) are genome-encoded bioactive molecules with broad-spectrum antibacterial and antitumor properties, playing a crucial role in innate immunity^7,8^. They exert antimicrobial effects through multiple mechanisms such as membrane disruption, biofilm inhibition, and interference with intracellular metabolism^9,10^. The artificial design of hybrid AMPs has emerged as a research focus, involving sequence optimization and structural modifications to enhance bioactivity, target specificity, stability, and reduce toxicity^11^. Parent peptides can originate from natural peptides, functional protein fragments, anticancer peptides, or cell-penetrating peptides^12^. Studies indicate that such modified AMPs often display improved efficacy while exhibiting lower toxicity and reduced synthesis costs^13^. Recent studies have also demonstrated effective antimicrobial interventions against Gram-negative pathogens such as *Escherichia coli* (*E. coli*), further supporting the feasibility of developing novel antimicrobial strategies with broad antibacterial applicability^14^. Developing novel AMPs and optimizing existing ones are therefore critical for overcoming current limitations and expanding their potential therapeutic applications.

Based on their primary modes of action, AMPs can be broadly classified into membrane-lytic peptides and intracellular-targeting peptides. Representative examples of these two mechanistic classes include SAAP-148 and Buforin IIb, respectively. SAAP-148, a synthetic derivative of the natural LL-37, has been optimized for enhanced antimicrobial activity and stability. It effectively targets resistant bacteria such as MRSA, *Carbapenem-Resistant Enterobacterales* (CRE), and *Vancomycin-Resistant Enterococcus* (VRE) by disrupting bacterial membrane integrity, leading to cell lysis^15^. Additionally, SAAP-148 exhibits strong biofilm penetration, making it a promising candidate for treating chronic wound infections^16^. In contrast, Buforin IIb, a synthetic derivative of Buforin II, penetrates bacterial membranes without causing significant lysis^17^. Once inside the cytoplasm, it interacts with nucleic acids, thereby inhibiting essential cellular processes^18^.

**Fig. 1:**
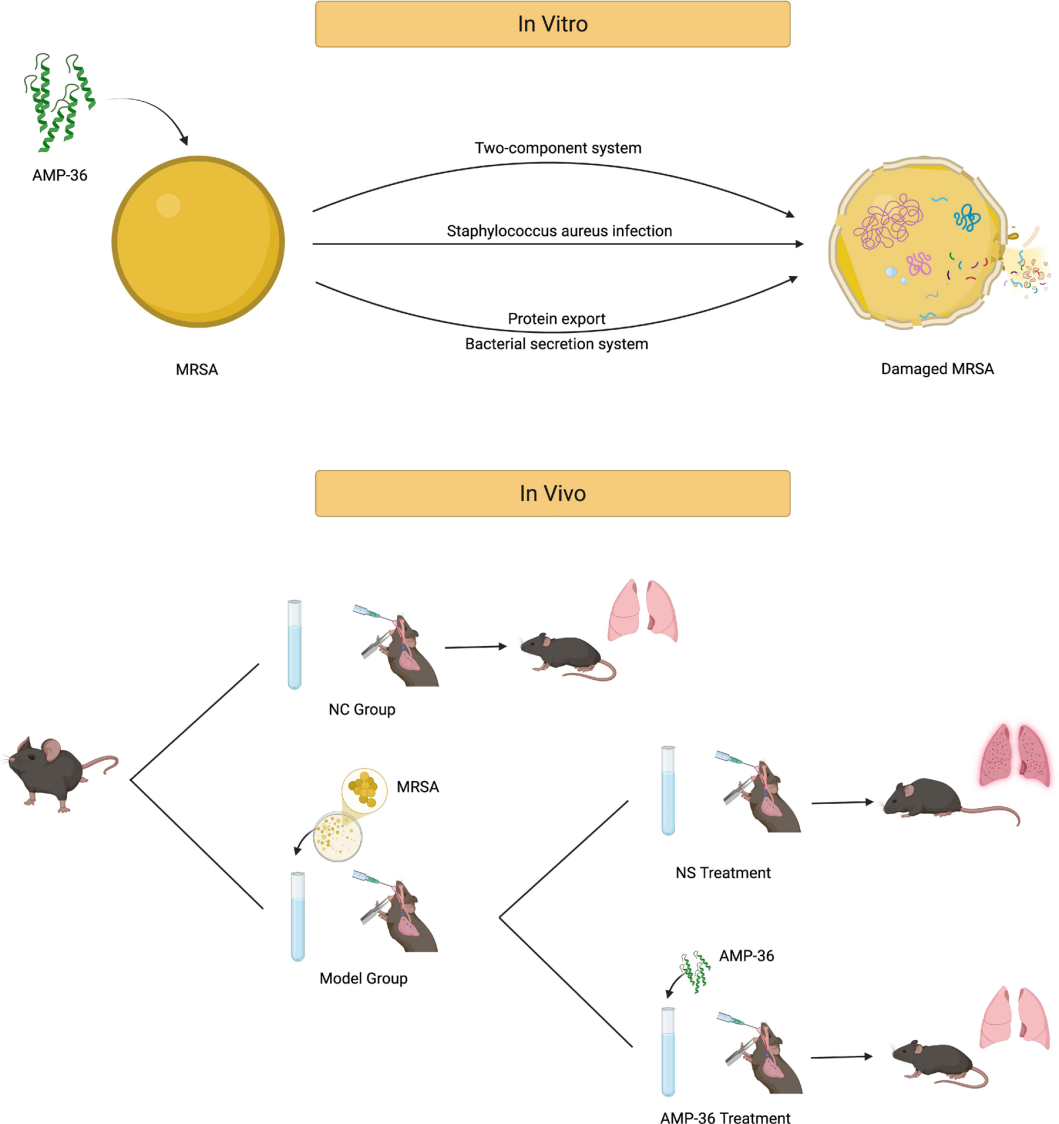
Overview of the study design and experimental line. Created with BioRender.com.

Based on the antimicrobial mechanisms of AMPs, this study aimed to synthesize a novel peptide that combines potent membrane-disrupting activity with intracellular targeting capability against drug-resistant bacterial strains, particularly MRSA. Furthermore, the in vivo anti-infective efficacy of this peptide was evaluated in a mouse model of pneumonia (Fig. 1). The findings may provide new insights into peptide development and offer a synergistic strategy for combating resistant bacterial infections.

## Results

### Characterization of AMP-36

**Fig. 2:**
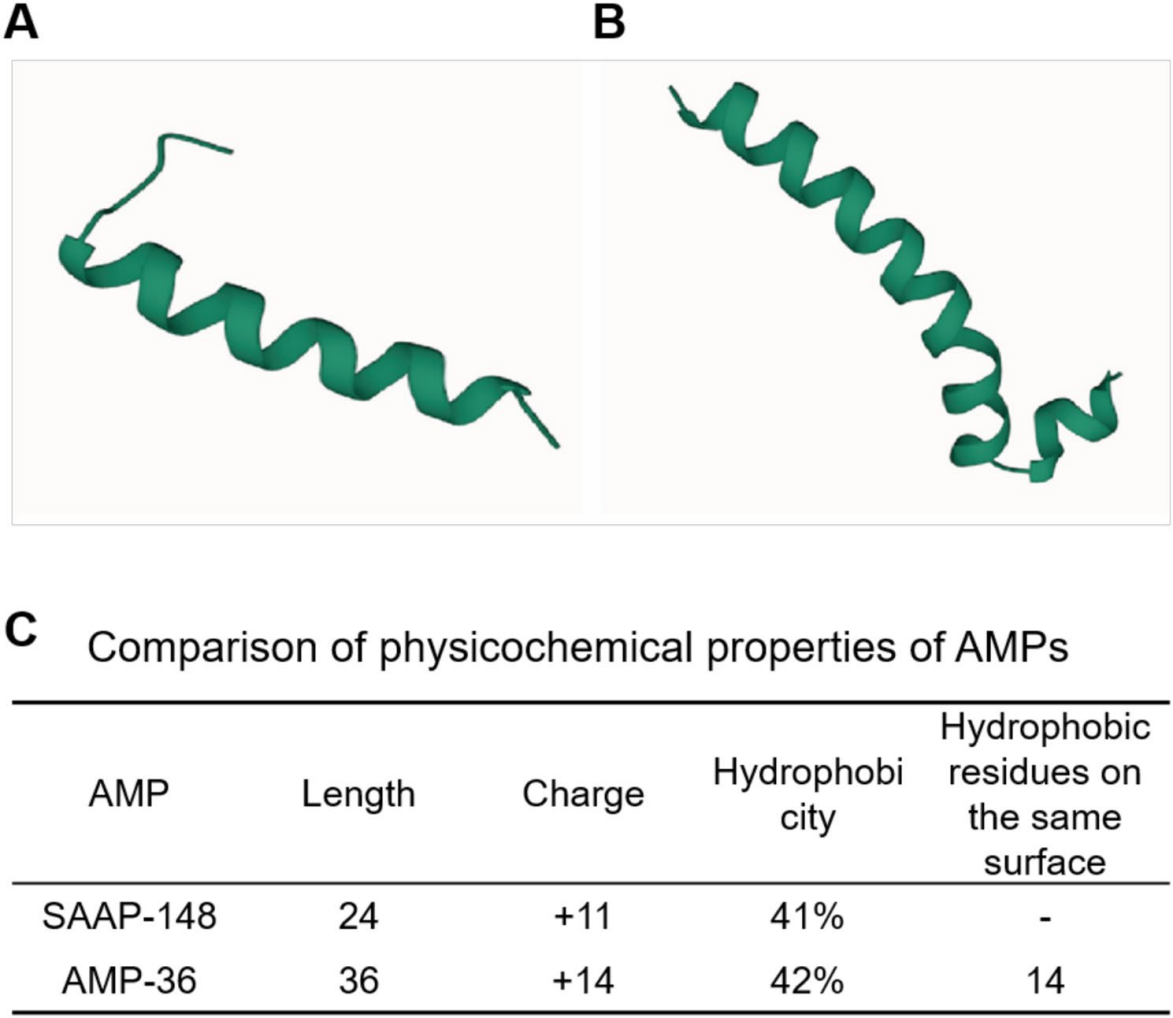
Structural prediction and physicochemical property characterization of SAAP-148 and AMP-36. (**A**) Predicted protein structures of SAAP-148 by AlphaFold. (**B**) Predicted protein structures of AMP-36 by AlphaFold. (**C**) Comparison of physicochemical properties of SAAP-148 and AMP-36 predicted by the DBAASP and APD3 databases.

Based on AlphaFold prediction, SAAP-148 forms a short and compact α-helix (Fig. 2A), whereas AMP-36 displayed a longer, more rigid α-helix (Fig. 2B). Physicochemical properties predicted by the DBAASP and APD3 databases further highlighted these differences (Fig. 2C). SAAP-148 consists of 24 amino acids, with a net charge of + 11 and hydrophobicity of 41%, though the distribution of hydrophobic residues on the same surface was not predicted. In contrast, AMP-36 was extended to 36 amino acids, exhibited an increased charge of + 14, slightly higher hydrophobicity of 42%, and featured 14 hydrophobic residues clustered on the same surface.

### AMP-36 exhibits broad-spectrum antibacterial activity

The bactericidal activity was assessed by determining the minimum inhibitory concentration (MIC). For the quality control strain, the MIC of SAAP-148 was 8 μg/mL, whereas that of AMP-36 was reduced to 4 μg/mL. Both peptides demonstrated antibacterial activity against clinically isolated MDR strains. Notably, AMP-36 showed stronger activity than its parent peptide SAAP-148 against MRSA, CRE, and MDR-Ab, while their antibacterial effects against CRKP were comparable (Table 1). In addition, AMP-36 also exhibited inhibitory activity against representative Gram-negative bacteria, including both ATCC strains and clinical isolates of *P. aeruginosa* and *E. coli* (Table S1), supporting its antibacterial activity across a broad range of bacterial species.

**Table 1:** MIC of quality control strain and clinical isolates.

Strains		MIC	
		SAAP-148	AMP-36
G +	*S. aureus* ATCC 29,123	8	4
	MRSA (240,302,491)	32	16
	MRSA (240,902,626)	32	16
	MRSA (240,902,714)	16	16
G-	CRE (240,202,447)	8	4
	CRKP (240,320,634)	16	16
	MDR-Ab (240,602,497)	8	4

*MIC: minimum inhibitory concentration (μg/mL). G + : Gram-positive bacteria; G-: Gram-negative Bacteria. Strains: Staphylococcus aureus (*S. aureus*), Methicillin-resistant *S. aureus* (MRSA), Carbapenem-resistant *Enterobacterales* (CRE), Carbapenem-resistant *Klebsiella pneumoniae* (CRKP), Multidrug-resistant *Acinetobacter baumannii* (DR-Ab).

### AMP-36 demonstrates rapid and concentration-dependent killing of MRSA in vitro

**Fig. 3:**
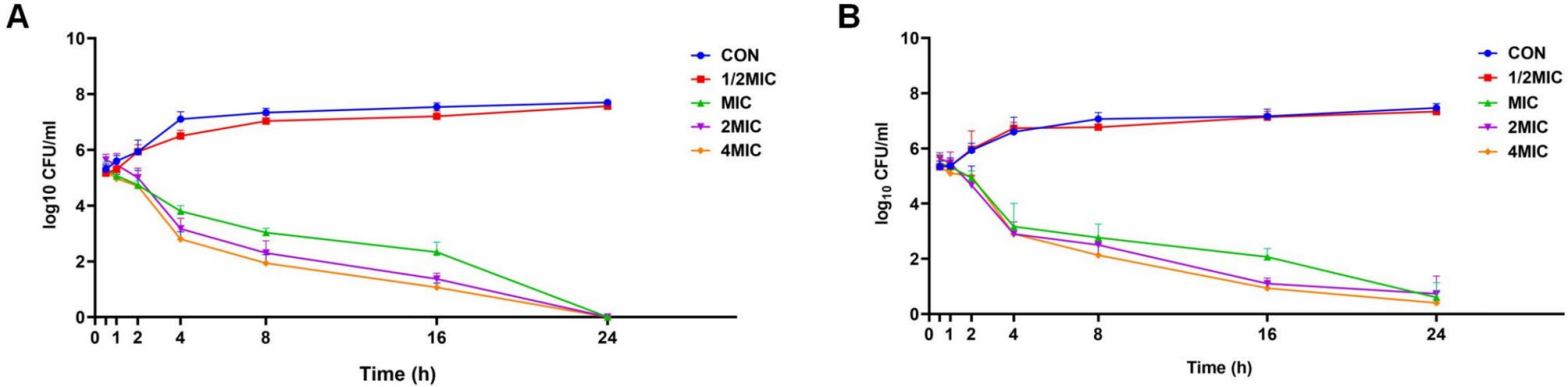
Time-kill curves of AMP-36 (**A**) and SAAP-148 (**B**) against MRSA over time at different multiples of respective MIC. MRSA suspensions (1 × 10^6^ CFU/mL) were incubated with AMPs at 0, 1/2 × , 1 × , 2 × , and 4 × MIC. Samples were collected at designated time points from 0 to 24 h, serially diluted, plated on LB agar, and CFU were counted after incubation for 18–20 h . Data are represented as mean ± SEM from three independent experiments.

A time-kill assay was conducted to evaluate the antibacterial activity of AMP-36 against MRSA at different multiples of the MIC over varying treatment durations. After 1 h of exposure, MRSA cultures treated with AMP-36 at concentrations ≥ 1 × MIC or higher MIC showed an approximate 1-log reduction in colony-forming units (CFU). Prolonged treatment for 24 h led to a marked decrease in CFU counts, reaching near-undetectable levels (Fig. 3A). In contrast, SAAP-148, the parental peptide, exhibited comparable bactericidal activity against MRSA but required a two-fold higher concentration. These findings demonstrate that AMP-36 exerts rapid and potent antibacterial effects against MRSA (Fig. 3B).

### AMP-36 shows therapeutic potential against MRSA pneumonia in mice

**Fig. 4:**
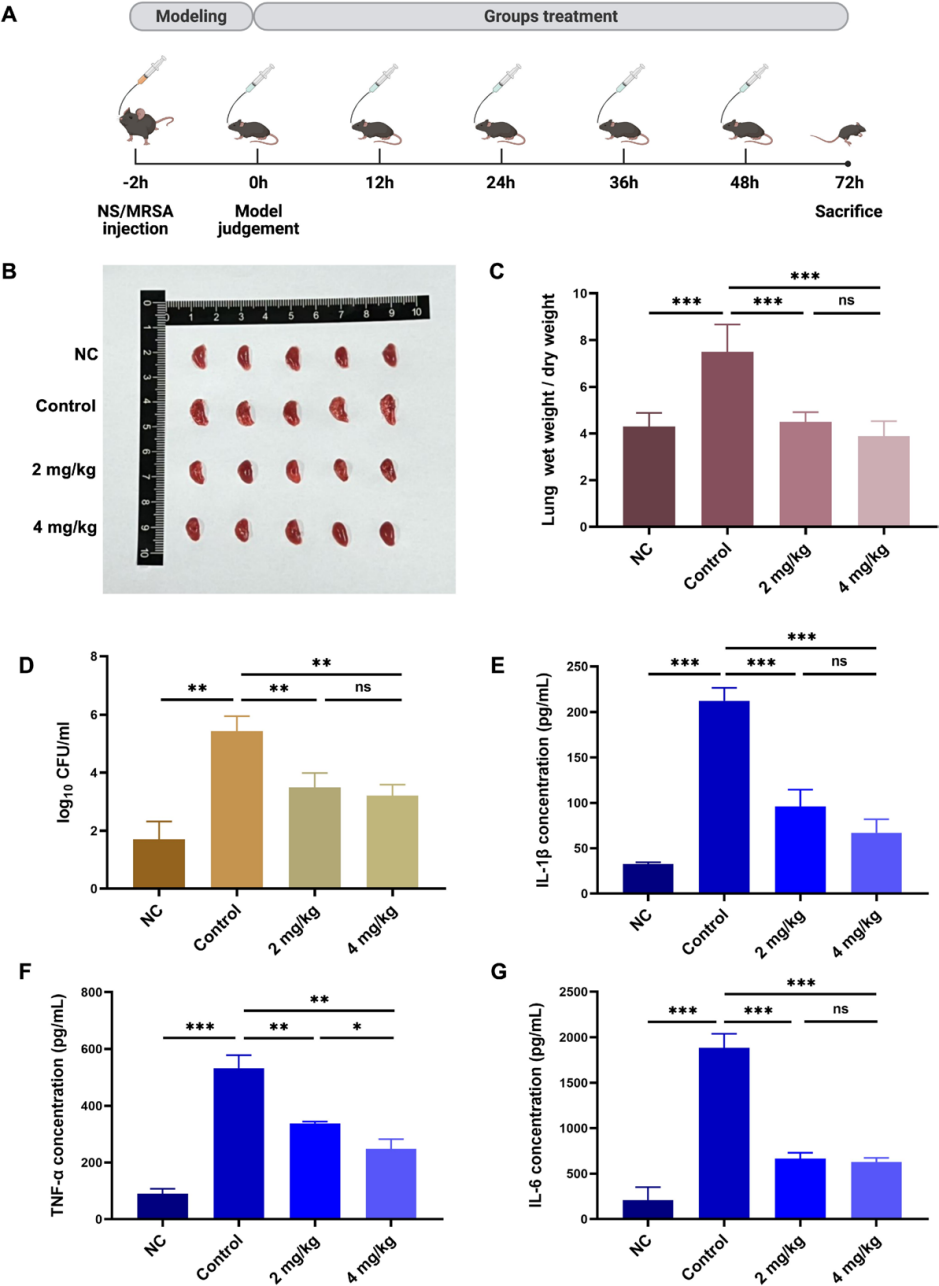
Therapeutic effects of AMP-36 in a mouse model of MRSA pneumonia. (**A**) Experimental timeline of the *in vivo *experiment. Mice were intratracheally inoculated with PBS or MRSA at 2 h post-infection, followed by AMP-36 treatment at 0 h, 12 h, 24 h, 36 h, 48 h. The mice were euthanized at day 3. (**B**) Images of right lungs of the mice from each group. (**C**) Lung wet weight/dry weight (W/D) ratio comparsions. (**D**) Bacterial load (log_10_ CFU/mL) in BALF. (**E–G**) Concentrations of *IL-1β* (**E**), *TNF-α* (**F**), and *IL-6* (**G**) in BALF, measured by ELISA. Data are presented as mean ± SEM from three independent experiments, analyzed by one-way ANOVA (ns: *p* > 0.05, ^*^p < 0.05, ^**^p < 0.01, ^***^p < 0.001). NC group: normal control; Control group; MRSA-infected mice without AMP-36 treatment; 2 mg/kg group: MRSA-infected and treated with 2 mg/kg AMP-36; 4 mg/kg group: MRSA-infected and treated with 4 mg/kg AMP-36 (n = 5 for each group). A was created with BioRender.com.

The above experiments investigated the bactericidal activity of AMP-36 against various clinically isolated bacterial strains, as well as its time-kill characteristics against MRSA in vitro. However, its in vivo antibacterial efficacy remains unclear. To address this, a mouse model of acute MRSA-induced lung infection was established to evaluate the in vivo antibacterial effects of AMP-36, following previously described procedures^19^ (Fig. 4A). After 4 rounds of treatments, mice in the treatment groups showed marked improvements in activity and overall condition. Compared with the control group, treated mice displayed more frequent spontaneous behaviors, such as cage exploration and active movement, along with quicker responses to external stimuli and restored food and water intake. These observations indicate that infection impaired the mental status of mice, while treatment effectively alleviated these symptoms. (Video S1).

### AMP-36 reduces pulmonary edema, bacterial load and inflammatory cytokine expression in BALF

The lung wet-to-dry (W/D) weight ratio, an indicator of pulmonary edema, was significantly reduced in AMP-36–treated mice compared with controls (Fig. 4B, C; *p* < 0.001, *p* < 0.001). However, no significant difference was observed between the 2 mg/kg and 4 mg/kg treatment groups (Fig. 4B, C; *p* > 0.05).

To assess bacterial clearance and infection control, both bacterial load and inflammatory factor expression in bronchoalveolar lavage fluid (BALF) were measured. Plate count analysis revealed that, compared with the control group, the treatment groups exhibited a significantly reduced bacterial load in BALF (Fig. 4D; *p* < 0.01). However, there was no significant difference in the bacterial load between the high-dose group and the low-dose group (Fig. 4D; *p* > 0.05). Compared with controls, mice treated with AMP-36 showed significantly reduced levels of *IL-1β*, *TNF-α*, and *IL-6* in BALF, as measured by ELISA. (Fig. 4E, F, G; *p* < 0.001,* p* < 0.01,* p* < 0.001).

### AMP-36 significantly decreased inflammatory response in lung tissue

**Fig. 5:**
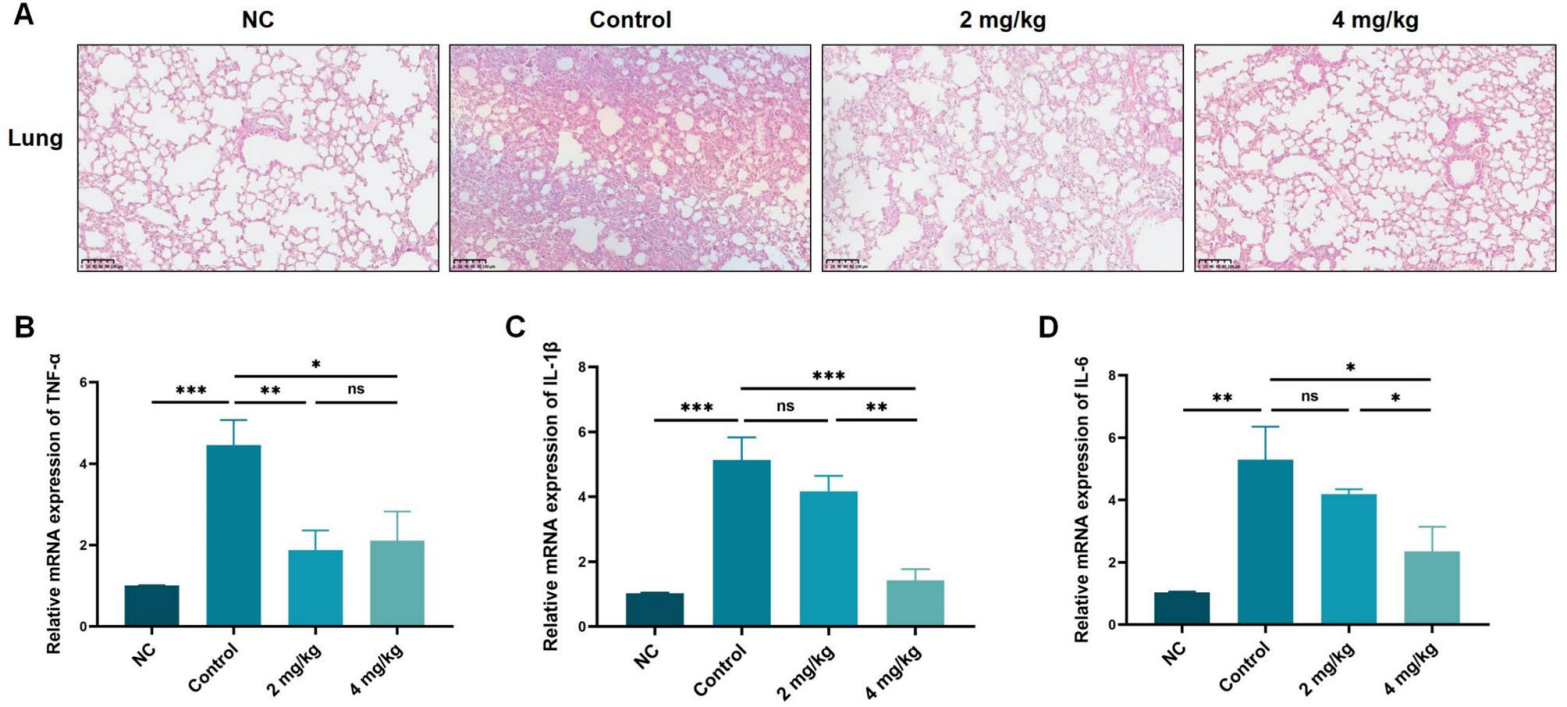
Lung tissue morphology and inflammatory factor expressions at endpoint (day 3). (**A**) HE staining of lung tissue of each group (scale bar = 100 µm). (**B-D**) mRNA expression of inflammatory cytokines *TNF-α* (**B**), *IL-1β* (**C**), and *IL-6* (**D**) in lung tissues, measured by qPCR (ns: no statistically signifance, *p* > 0.05), ^*^p < 0.05, ^**^p < 0.01, ^***^p < 0.001).

To evaluate the severity of the inflammatory response in lung tissue, Hematoxyin and Eosin (HE) staining was performed. The results showed that treatment with both doses (2 mg/kg and 4 mg/kg) of AMP-36 markedly alleviated pathological damage, with reduced inflammatory cell infiltration, milder interstitial edema, and less congestion (Fig. 5A). mRNA expression of the inflammatory factors in lung tissues was quantified by qPCR. Treatment with both doses of AMP-36 significantly reduced *TNF-α* expression (Fig. 5B; p < 0.01, *p* < 0.05). Notably, lung tissues from mice treated with 4 mg/kg showed significantly reduced *IL-1β* (Fig. 5C; *p* < 0.01) and *IL-6* (Fig. 5D; *p* < 0.05) compared with controls, whereas the lower dose had no significant effect.

### AMP-36 damaged membrane of MRSA

**Fig. 6:**
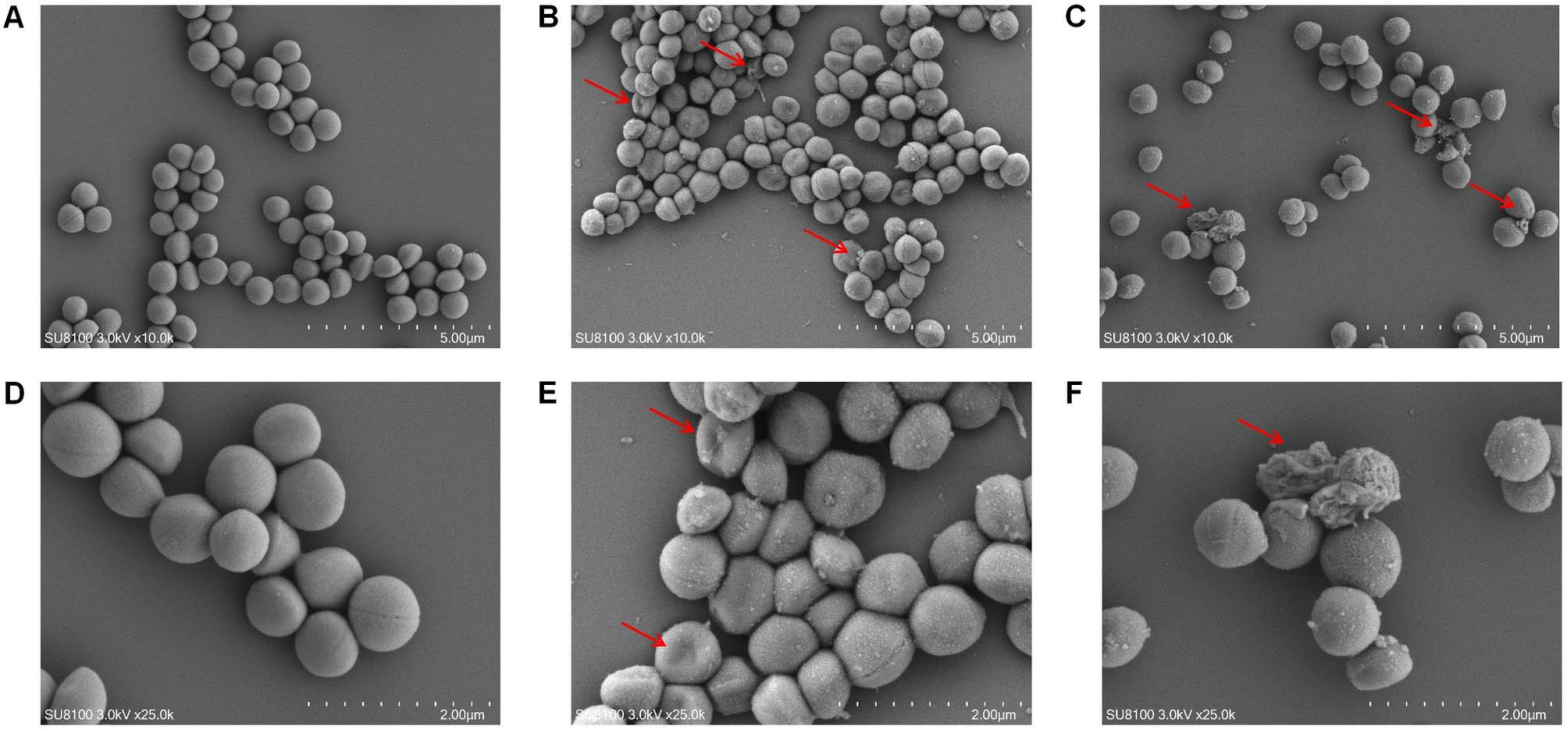
SEM images of MRSA exposed to AMP-36 without treatment (**A, D**), with 1 × MIC (**B, E**) and with 2 × MIC (**C, F**) for 2 h. **A-C** show representative images captured at a magnification of 10,000 × , **D-F** show higher-magnification images at 25,000 × . Red arrows indicate membrane disruption, surface deformation, and leakage of intracellular contents induced by AMP-36.

Scanning electron microscopy (SEM) characterization was performed on MRSA treated with AMP-36 at 1 × MIC and 2 × MIC for 2 h. The SEM image revealed distinct morphological changes in MRSA following AMP-36 treatment. In the control group, MRSA appeared as a regular sphere with a smooth surface and intact morphology (Fig. 6A, D). After exposure to 1 × MIC of AMP-36, the bacterial surface showed signs of shrinkage and deformation (Fig. 6B, E). Treatment with 2 × MIC caused even more pronounced damage, with severely disrupted cell structures and evident leakage of intracellular contents (Fig. 6C, F).

### mRNA sequencing revealed that AMP-36 exerts bactericidal effects against MRSA through multiple pathways.

**Fig. 7:**
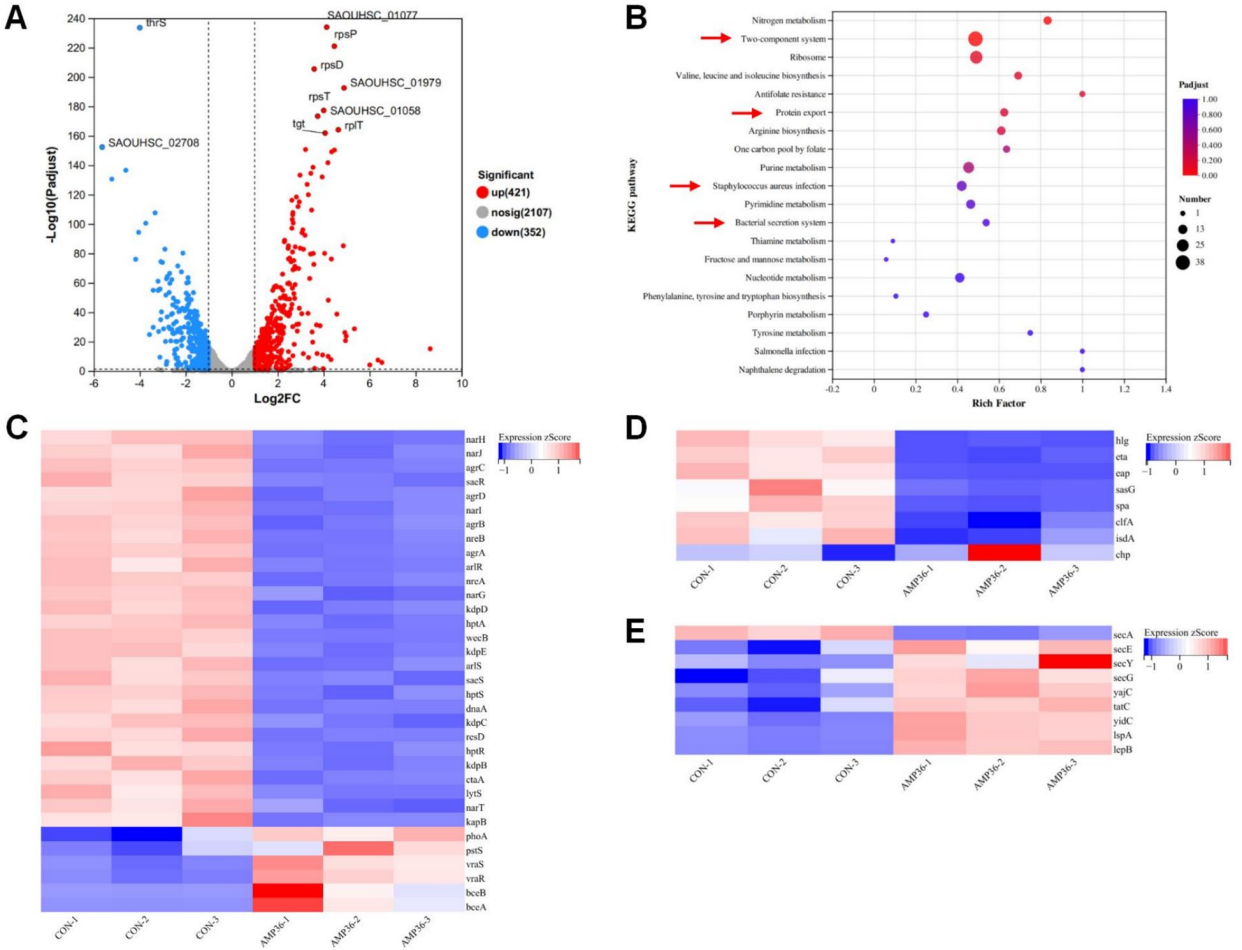
Transcriptomic analysis of MRSA treated with AMP-36 after 2 h. (**A**) Volcano plot of DEGs between AMP-36-treated MRSA and untreated controls (|log2FC|> 0, *p* < 0.05). (**B**) KEGG pathway enrichment analysis of DEGs. (**C**) Heatmap of TCS pathway. (**D**) Heatmap of *Staphylococcus aureus* infection pathway. (**E**) Heatmap of Protein export and Bacterial secretion system pathways. Data were obtained from three independent biological replicates. Pathway image adapted from KEGG (Kyoto Encyclopedia of Genes and Genomes), Kanehisa Laboratories.

Comprehensive transcriptomics analysis was conducted to elucidate the anti-MRSA mechanism of AMP-36. Verification of RNA integrity and uniform expression distribution confirmed data reliability (Figure S1A, Figure S1B). Differentially expressed genes (DEGs) in MRSA from each group were identified and their expression levels were analyzed. Compared with the control group, 773 DEGs were detected under AMP-36 treatment at the MIC level, including 421 up-regulated and 352 down-regulated genes (Fig. 7A). To further elucidate the functional roles and biological significance of these DEGs, Gene Ontology (GO) analysis was performed. DEGs were predominantly enriched in pathways associated with inosine monophosphate (IMP) biosynthesis and metabolism, while also perturbing the nucleoside phosphate metabolic process and the reactive nitrogen species metabolic process. In addition, significant interference with the nitrate metabolic process was observed, collectively exerting broad impacts on fundamental biological processes (Figure S2). The KEGG database is a comprehensive resource for biological pathways and molecular interaction networks^20–22^. Based on the KEGG pathway database, the TOP 20 enriched pathways were shown in the Bubble Plot. Among these, four pathways associated with the virulence of MRSA were identified: Two-component system (TCS), *Staphylococcus aureus* infection, Protein export, and the Bacterial secretion system (Fig. 7B). In the Two-component system pathway, key DEGs such as *agr* (accessory gene regulator), *saeRS* (*Staphylococcus aureus* exoprotein expression), and *arlRS* (DNA-binding response regulator) were significantly downregulated following AMP-36 treatment, whereas others, including *vraRS* (Vancomycin-resistance associated sensor/regulator) were significantly upregulated (Fig. 7C). Within the *Staphylococcus aureus* infection pathway, critical virulence-associated DEGs such as *glh* (γ-hemolysin), *eta* (enterotoxin A), *spa* (protein A), and *clfA* (clumping factor) were significantly downregulated (Fig. 7D). Furthermore, the Protein export and Bacterial secretion system pathways exhibited substantial with the key DEGs *secA* (accessory Sec system translocase SecA2) showing a clear downregulation trend (Fig. 7E). To validate the accuracy of the RNA-Seq data, eight genes related were selected from the analyzed DEGs and examined by qPCR. Results showed a strong correlation with the corresponding mRNA expression profiles obtained from RNA-Seq (Figure S3), thereby confirming the reliability of the transcriptomic data.

### AMP-36 exhibited low hemolytic activity in vitro and caused no observable organ damage in vivo

**Fig. 8:**
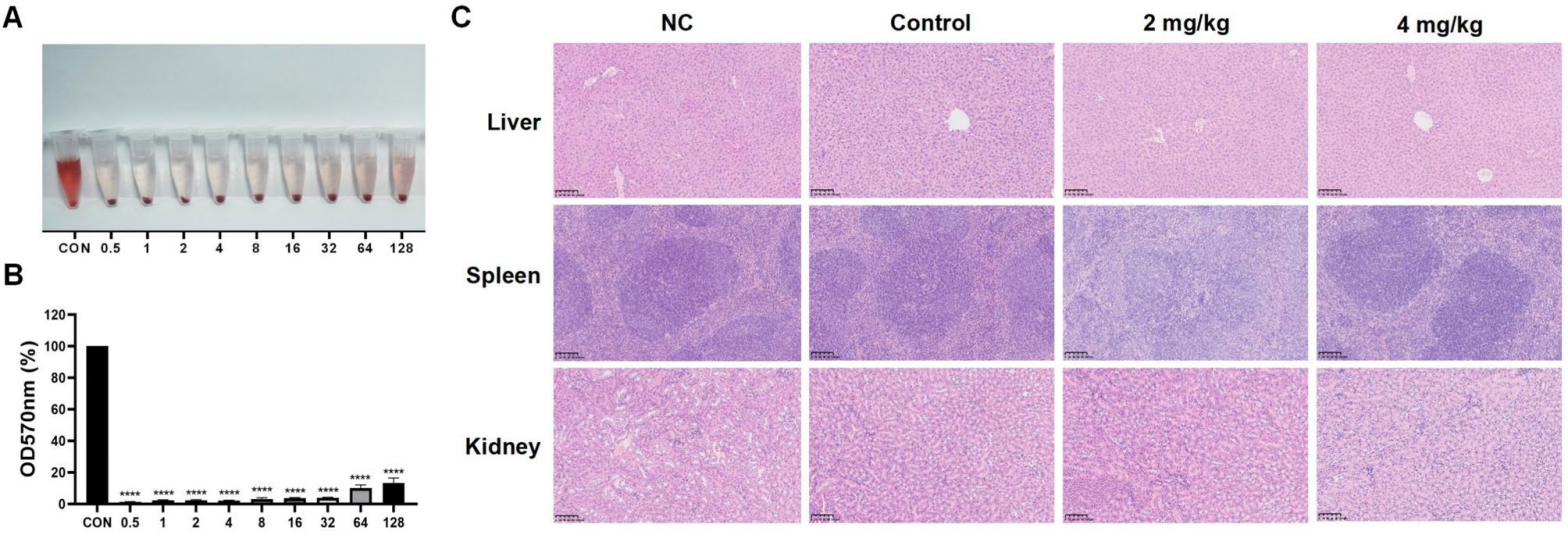
Toxicity evaluation of AMP-36. (**A**) Visual representation of hemolysis assay of red blood cells after incubation with AMP-36 at the concentrations indicated (0.5–128 μg/mL) for 1 h. Sterile PBS was used as negative control for baseline correction and ddH_2_O as positive control defining complete hemolysis. (**B**) Hemolysis rates measured by OD570 nm. Data are presented as mean ± SEM from three independent experiments, analyzed by one-way ANOVA (^****^p < 0.0001). (**C**) Representative HE-stained sections of liver, spleen, and kidney tissues from mice in each group at the experimental endpoint (scale bars = 100 μm).

The in vitro biocompatibility of AMP-36 was conducted using a hemolysis assay. AMP-36 induced concentration-dependent hemolysis, with the extent of red blood cell lysis increasing proportionally to the treatment concentration (Fig. 8A). At its MIC of 16 μg/mL, AMP-36 exhibited a hemolytic rate of < 5% (OD 570 nm), which was comparable to that of the negative control (Fig. 8B, p < 0.0001). At higher concentrations, such as 128 μg/mL, a slight but measurable increase in hemolysis was observed (Fig. 8B). Importantly, the hemolysis profile did not show a sharp or disproportionate increase across the tested concentration range, suggesting that no overt alteration in erythrocyte osmotic stability was observed under the experimental conditions.

HE staining was performed to assess the potential toxicity of AMP-36 on vital organs. In AMP-36-treated mice, the liver displayed intact architecture with no signs of necrosis, inflammatory infiltration, or sinusoidal dilation compared with infected controls. The morphology of Splenic white and red pulp remained normal across all cohorts. Histological examination of the kidneys revealed no evidence of necrosis or structural damage (Fig. 8C).

## Discussion

MDR bacterial infection poses a major threat in modern medicine and are a leading cause of healthcare-associated infections^23^, and pneumonia represents a common and severe manifestation caused by MDR bacteria. Immunocompromised patients, especially those with hematological cancer patients who have undergone radiation therapy or chemotherapy, are at the highest risk for severe MRSA infections^24^. AMPs have recently emerged as a promising alternative to conventional antibiotics for bacterial eradication, owing to their potent antibacterial activity and low likelihood of inducing resistance ^25^.

In this study, we designed a novel peptide, AMP-36, using sequence optimization strategies targeting genetic materials. Artificial intelligence (AI) and machine learning (ML) tools are increasingly being applied to the rational design and synthesis of lead peptides^26^. AlphaFold, a deep-learning-based platform, accurately predicts three-dimensional protein structures from amino acid sequences^27^. Structural optimization, particularly of α-helices, is crucial for enhancing AMP efficacy^28^. In this study, AF revealed that SAAP-148 forms a relatively flexible short α-helix, which allows rapid but limits deeper membrane penetration and stability. Besides, physicochemical properties significantly influence AMP bioactivity. Database tools such as DBAASP (www.dbaasp.org/) and APD3 (http://aps.unmc.edu/AP/) have proven valuable for predicting AMP properties. Comparative analyses indicated that AMP-36 possesses an extended amino acid sequence, increased positive charge, and higher hydrophobicity. These structural advantages likely enhance electrostatic binding, membrane insertion, and structural stability, collectively improving antibacterial activity^29,30^.

Subsequently, in vitro functional validation was conducted to characterize the biological activities of AMP-36. Using ATCC and multiple clinical MDR isolates, antimicrobial assays demonstrated that AMP-36 exhibited potent antimicrobial activity and rapid bactericidal activity, achieving near-complete bacterial clearance within 8 h. Such rapid killing characteristic are a desirable property for antimicrobial agents intended for the treatment of acute and severe infections, where prompt control of bacterial burden is critical. MRSA is increasingly implicated in severe infections, contributing substantially to bloodstream infections, pneumonia, surgical site infections. As a leading cause of hospital-acquired pneumonia, MRSA infection is associated with high morbidity and mortality rates^31^, highlighting the importance of in vivo studies using MRSA pneumonia animal models. In vivo, AMP-36 treatment significantly reduced lung edema, decreased bacterial loads in BALF, and attenuated inflammatory exudation. These effects are likely attributed to efficient bacterial clearance, which subsequently alleviates infection-associated inflammatory responses. Consistent with the activity of other AMPs, AMP-36 reduced pro-inflammatory cytokine levels in both BALF and lung tissues, suggesting a potential to mitigate host inflammatory damage secondary to bacterial burden reduction. This effect may be mediated by reduced release of pathogen-associated molecular patterns (PAMPs) following bacterial clearance^32^. It should be noted that only selected pro-inflammatory cytokines (*TNF-α*, *IL-1β*, and *IL-6*) were measured in this study, and the involvement of other cytokines and chemokines was not evaluated, which represents a limitation and warrants further investigation. Various studies reveal that drug-induced bacterial inactivation involves different mechanisms. Graphene quantum dots (GQDs) disrupt *E. coli* biofilm formation and interfere with sulfur and nitrogen metabolism^33^. Likewise, X33 antimicrobial oligopeptide (X33 AMOP) restrains Penicillium digitatum by damaging cell integrity, interfering with genetic information processes, decreasing oxidative stress tolerance, and disrupting energy metabolism^34^. In addition to antibacterial efficacy, biosafety represents a critical consideration for the clinical translation of AMPs. Hemolysis assays demonstrated that AMP-36 induced minimal erythrocyte toxicity, even at concentrations substantially exceeding its MIC, indicating favorable in vitro biocompatibility. Furthermore, histological examination of major organs by HE staining revealed no significant pathological alterations in AMP-36 treated mice. Together, these findings suggest that AMP-36 possesses a preliminary safety profile compatible with in vivo application. These findings emphasize the complex nature of antimicrobial actions.

To further elucidate the mechanism of AMP-36-mediated MRSA killing, SEM and RNA-seq analysis were performed. SEM analysis revealed that AMP-36 induces rapid and severe disruption of bacterial membrane integrity at bactericidal concentrations, indicating that AMP-36 exerts its bactericidal effect primarily by disrupting the integrity of the bacterial cell membrane. Under such conditions, bacterial cell lysis and loss of homeostasis are expected to result in widespread transcriptional dysregulation. Accordingly, RNA-seq analysis revealed downregulation of genes involved in purine biosynthesis, virulence factor production, and secretion systems following AMP-36 treatment. Importantly, these transcriptional changes are more likely secondary consequences of membrane disruption and cellular collapse.

Notably, purine nucleotides, such as ATP and GTP, are essential for multiple critical cellular functions in bacteria. Within the de novo purine biosynthesis pathway, inosine monophosphate (IMP) serves as a key precursor for the synthesis of all purine nucleotides, and its production is indispensable for nucleic acid biosynthesis, energy metabolism, and the generation of cofactors that mediate vital metabolic reactions^35^. The *pur* gene family encoding key enzymes involved in the de novo IMP biosynthesis^36^, was significantly downregulated, which is expected to impair de novo IMP synthesis. The suppression of purine biosynthetic genes is therefore consistent with global metabolic failure following membrane disruption, rather than direct inhibition of the purine synthesis machinery.

Importantly, the pathogenicity of MRSA is largely driven by its virulence factors, most of which are secreted via specialized secretion systems^37^. RNA-seq analysis also revealed marked downregulation of virulence-associated genes, including *hlg*, *lukS*, *lukF*, *eta*, *spa*, *clfA*, and *sasG*, *isdA*, which encode major toxins and surface proteins critical for hemolysis, leukocyte killing, immune evasion, and adhesion to host tissues^38^. The reduced expression of these virulence factors likely reflects a downstream consequence of cellular stress and impaired regulatory capacity following membrane damage. Although *chp* (the chemotaxis-inhibiting protein) was upregulated, this response appears to represent a limited compensatory stress reaction that is outweighed by the broad suppression of major virulence determinants.

Alterations were also observed in genes associated with two-component systems (TCS). The TCS serves as a crucial signaling pathway for bacterial response to environmental stress^39^. Among DEGs in TCS, *agr* system regulates the quorum sensing network, and together with the *saeRS* coordinates the expression of toxins, such as α-hemolysin, enterotoxins, and protein A^38^. *ArlRS* is essential for adhesion, biofilm formation and virulence^40^. *KdpABC* encodes an oligomeric K^+^ transport complex that maintains ion homeostasis under stress conditions and directly interacts with the promoter of virulence genes^41^. And *dnaA* is a protein initiates chromosome replication. The downregulation of these systems further supports the notion that AMP-36-induced membrane damage disrupts bacterial signal transduction and regulatory networks. In contrast, the upregulation of *vraRS* (cell wall stress sensor) likely reflects a direct stress response of MRSA to membrane perturbation rather than a targeted regulatory effect.

In addition, genes involved in protein secretion and export pathways were downregulated following AMP-36 treatment. Interference with the bacterial secretion system and protein export pathways might affect the secretion and localization of virulence factors, thereby reducing the pathogenicity of MRSA. *SecA,* an ATP-driven protein, encodes a critical component of the general secretory pathway. It is an essential machinery required for the transmembrane of the precursor proteins^42,43^. Its reduced expression is expected to impair the secretion and surface localization of virulence factors, preventing key virulence factors such as *hla, clfA, and eap* from being effectively delivered to the bacterial cell surface or extracellular environment, thereby further diminishing pathogenicity. Again, this effect is most consistent with secondary consequences of energetic collapse and stress signaling induced by membrane disruption.

Although GO and KEGG analyses revealed enrichment of multiple biological pathways, pathway enrichment reflects global transcriptional responses. Based on SEM evidence, membrane disruption is likely the primary antibacterial action of AMP-36. The altered expression of virulence and metabolic genes is therefore more reasonably attributed to secondary stress responses following membrane damage rather than direct targeting of specific intracellular pathways. Whether AMP-36 exerts additional direct intracellular effects remains to be determined and will require further mechanistic studies.

In summary, AMP-36 exhibits potent antibacterial activity against MRSA and demonstrates effective therapeutic efficacy in a murine model of MRSA-induced pneumonia, highlighting its potential as a promising alternative for the treatment of hospital-acquired MRSA infections. Several limitations of this study should be acknowledged. Based on online structural prediction, AMP-36’s structural stability in bacterial cells has not been experimentally verified and remains to be determined. Besides, while AMP-36 was rationally designed from SAAP-148 and exhibited improved in vitro antibacterial activity, comparative evaluation of their in vivo therapeutic performance will be important to further clarify the translational impact of the structural modifications. In addition, the in vivo dosing strategy was based on MIC-guided scaling, further studies are required to evaluate long-term safety, particularly with respect to systemic exposure and repeated administration. And the therapeutic efficacy of AMP-36 was not directly compared with positive antibiotic control such as vancomycin or linezolid. Therefore, the in vivo findings should be interpreted as the therapeutic activity relative to no treatment. Finally, transcriptomic changes observed following AMP-36 treatment are more likely to represent secondary stress responses to membrane disruption rather than direct intracellular targeting, and the underlying mechanisms require further investigation.

Conclusively, the results of our study support the hypothesis that rationally designed antimicrobial peptide AMP-36 can achieve effective antibacterial activity primarily through membrane-associated mechanisms, thereby offering therapeutic potential against MRSA infections. The findings of this work provide a conceptual framework for the further development of AMP-36 as an antimicrobial candidate. Future work will focus on deeper mechanistic investigations to further delineate the membrane-associated antibacterial actions of AMP-36 and to determine whether any additional intracellular effects contribute to its activity. In parallel, rational optimization of AMP-36 and related peptide analogues, guided by structure–activity relationships and safety profiling, may facilitate the development of next-generation AMP candidates with improved efficacy and translational potential.

## Materials and methods

### Acquisition, activation and preservation of bacterial strains

The bacterial strains were provided by the Microbiology Laboratory Department of the Second Hospital of Shandong University. The frozen strains were streaked on fresh Luria–Bertani (LB) agar plates (L8290, Solarbio, China) and incubated at 37 °C. Single colony forming units (CFU) were picked and transferred to LB broth medium (L8291, Solarbio, China) and cultured under 220 rpm, 37 °C shaker conditions for subsequent experiment. For long-term storage, bacterial cultures were stored at − 80 °C in LB broth supplemented with 25% (v/v) sterile glycerol as a cryoprotectant.

### Design, analysis and synthesis of AMPs

AMP-36 was rationally designed based on the sequence of SAAP-148 with the aim of optimizing antibacterial activity while reducing potential cytotoxicity. Design considerations included peptide length, net charge, hydrophobicity, and amphipathic distribution. Physicochemical properties were analyzed using the DBAASP (https://dbaasp.org/search) and APD3 databases (https://aps.unmc.edu/AP/). Structures were predicted using AlphaFold website (https://alphafoldserver.com/). The amino acid sequences of peptides were shown in Table 2. Peptides were synthesized by GL Biochem (Shanghai, China) using solid-phase Fmoc chemistry, followed by purification via high-performance liquid chromatography (HPLC) with UV detection to a purity of > 95%, and molecular weight confirmation was performed by liquid chromatography–mass spectrometry (LC–MS) (Figure S4). These analyses were used to support structural and functional interpretation of AMP-36.

**Table 2:** Amino acid sequences of AMP-36 and its parent peptide SAAP-148.

Peptide name	Amino acid sequence
SAAP-148	LKRVWKRVFKLLKRYWRQLKKPVR
AMP-36	LKRVWKRVFKLLKRYWRQLKKPVRRAGLQFPVGRLR

### Minimum inhibitory concentration (MIC)

MICs were determined according to the Clinical and Laboratory Standards Institute (CLSI) guidelines (M100) (https://clsi.org/shop/standards/m100/). Peptides were initially dissolved in sterile ddH_2_O to prepare the stock solution (1 mg/mL), and the stock solution was diluted into 256 μg/mL and then subsequently serially two-fold diluted in Mueller-Hinton broth (MHB) to obtain the desired working concentrations (256, 128, 64 to 0.5 μg/mL). For MIC determination, 100 μL of each peptide dilution was mixed with 100 μL of 1 × 10^6^ CFU/mL bacterial suspension in the 96 well plate (FPT016, Beyotime, China) in sequence, resulting in a final bacterial inoculum of 5 × 10^5^ CFU/mL per well. Wells containing bacteria and culture medium without peptide were used as growth controls (negative controls), and wells containing culture medium alone were used as blank controls. Next, Plates were incubated at 37℃ for 16–20 h. MIC values were defined as the lowest peptide concentration at which no visible bacterial growth was observed. All assays were performed at least in triplicate.

### Time‑dependent killing curve

MRSA bacterial culture was adjusted to an initial concentration of 1 × 10^6^ CFU/mL and incubated with AMPs at 37℃. At predetermined time points (0, 1, 2, 4, 6, 8, 12, and 24 h), aliquots were collected and diluted in series with sterile PBS, then plated onto LB agar plates. After incubating plates at 37℃ for 16–20 h, CFUs were enumerated and time-killing curves were constructed by plotting log10 CFU/mL versus time. All experiments were performed in triplicate.

### Hemolysis assay

Human blood cells were obtained from healthy volunteer donors. Written informed consent for blood donation and research use was obtained from all donors in the study. The collection and use of human samples were approved by the Ethics Committee of the Second Hospital of Shandong University (Approval No. KYLL.2024SCR001). All procedures involving human participants were conducted in accordance with the Declaration of Helsinki. Washed red blood cells were diluted with sterile saline to a final concentration of 2% (v/v) and incubated with AMP-36 at the indicated concentrations. AMP solutions were prepared at final concentrations. Sterile PBS was included as a negative control to determine background absorbance and was used for baseline correction in the calculation of hemolysis rates. And positive control (ddH_2_O) defined complete hemolysis. After 1 h incubation, samples were centrifuged (3000 rpm, 5 min, 4℃) and 100 µL supernatant was measured at a wavelength of 570 nm. Hemolysis (%) was calculated as: (Sample OD − Negative OD) / (Positive OD − Negative OD) × 100%.

### In vivo

Animal experiments were approved by the Ethics Committee of the Second Hospital of Shandong University (Approval No. KYLL.2024SCR001), in accordance with the ARRIVE guidelines (https://arriveguidelines.org). All experiments were performed in accordance with relevant guidelines and regulations. SPF-grade male C57BL/6 mice (6–8 weeks) were purchased from Huafukang Biotechnology Co., Ltd. (Beijing, China). Mice were randomly divided into 4 groups: NC, Control, AMP-36 (2 mg/kg), and AMP-36 (4 mg/kg) groups, 5 mice per group. The in vivo doses of AMP-36 (2 mg/kg and 4 mg/kg) were selected based on its in vitro antibacterial potency and the bacterial inoculum used in the murine infection model. The dosing strategy aimed to achieve systemic exposure similar to the corresponding MIC by defined multiples, a commonly used approach for initial in vivo efficacy evaluation. Mice were anaesthetized with isoflurane prior to all procedures. Firstly, a sterile tracheal intubation was performed, and 20 µL, 1 × 10^9^ CFU/mL of NS or MRSA suspension was instilled into the trachea. Treatment started 2 h post-infection and was repeated every 12 h for a total of four times. At the experimental endpoint, mice were euthanized by cervical dislocation in accordance with the AVMA Guidelines for the Euthanasia of Animals (2020).

### Lung macroscopic specimen and wet-to-dry ratio

On the 3^nd^ day, the end point of the experiment in vivo, the right lung of each mouse was excised and rinsed with saline. Then the color, texture, and size were then examined and photographed, and the wet weight (WW) was recorded. The tissue was subsequently dried at 60 °C for 48 h, after which the dry weight (DW) was measured. The wet-to-dry ratio was calculated as W/D weight.

### Bronchoalveolar lavage fluid (BALF)

After 5 times treatment on each group, mice were deeply anesthetized and the trachea was exposed, followed by endotracheal tube insertion and ligation. 500 μL of sterile PBS was slowly injected, followed by gentle chest massage for 30 s, and then the lavage fluid was withdrawn. This process was repeated three times to collect BALF. 100 μL of BALF was spread on LB agar plates, the remaining BALF was centrifuged, and the supernatant was collected to analyse the inflammatory cytokines. Inflammatory cytokine concentrations were measured using *IL-1β* (EK201B, Liankebio, China), *IL-6* (EK206, Liankebio, China), and *TNF-α* (EK282, Liankebio, China) ELISA kit.

### qPCR

Lung tissues were quickly collected, snap-frozen in liquid nitrogen, placed in RNase-free tubes, and stored at − 80 °C. Total RNA was extracted from mouse lung tissues using an RNA extraction kit (AG21017, Accurate Biotechnology Co.,Ltd, China), and the RNA was reverse transcribed into cDNA (11155ES10, Yeasen, China). Then, qPCR was used to measure mRNA expression levels.

### Hematoxylin and Eosin (HE) Staining

Tissues from each group were immediately fixed in 4% paraformaldehyde for 48 h. The tissues were then dehydrated, cleared, and embedded in paraffin. Sections were prepared and mounted on slides, followed by staining using HE staining kit (C0105M, Beyotime, China) to assess inflammation.

### Scanning electron microscope (SEM)

Co-cultured AMP solution with MIC concentration and 1 × 10^6^ CFU/mL bacterial suspension, and incubated. Collected the bacterial cells by centrifugation and fixed them with 2.5% glutaraldehyde for 2 h. After washing with PBS, a series of ethanol were dehydrated. After drying at the critical point, sprayed gold treatment. SEM was used to observe changes in bacterial morphology and to evaluate disruptive effects of AMPs on bacterial cell membranes.

### Transcriptome Sequencing

Bacterial suspension (1 × 10^6^ CFU/mL) was co-incubated with the AMP-36 for 2 h, followed by centrifugation to collect bacterial cells. RNA quality control, library construction and sequencing were done by Cosmos Wisdom Biotech Co., Ltd. on the Illumina NovaSeq 6000 platform. Significantly DEGs (|log2FC|> 1, *p* < 0.05) were identified and subjected to GO functional enrichment and KEGG pathway enrichment analyses to elucidate the potential molecular mechanisms underlying bactericidal activity of AMP-36. Key DEGs were validated by qPCR.

### Statistical analysis

All data are presented as means ± standard error (SEM). GraphPad Prism 8 (La Jolla, CA, USA) software was used to assess the differences between groups using a *t*-test and one-way analysis of variance; *p* < 0.05 was considered statistically significant.

## Supplementary Information

Supplementary Information 1.

Supplementary Information 2.

Supplementary Information 3.

## Data Availability

The RNA-seq datasets generated during this study have been deposited in the NCBI Sequence Read Archive (SRA) under the BioProject accession number PRJNA1433395. All datasets produced or analyzed during this study can be obtained from the corresponding author upon reasonable request.
