# Supplementary material for: AMP-36 exhibits potent therapeutic efficacy against MRSA pneumonia through membrane-target mechanism
Source: Sci Rep. 2026 Mar 17;16:13799. doi: 10.1038/s41598-026-44156-6 (PMC13128947; doi:10.1038/s41598-026-44156-6)
Supplement: Supplementary file 1 — Supplementary Information 1. [file 41598_2026_44156_MOESM1_ESM.pdf]

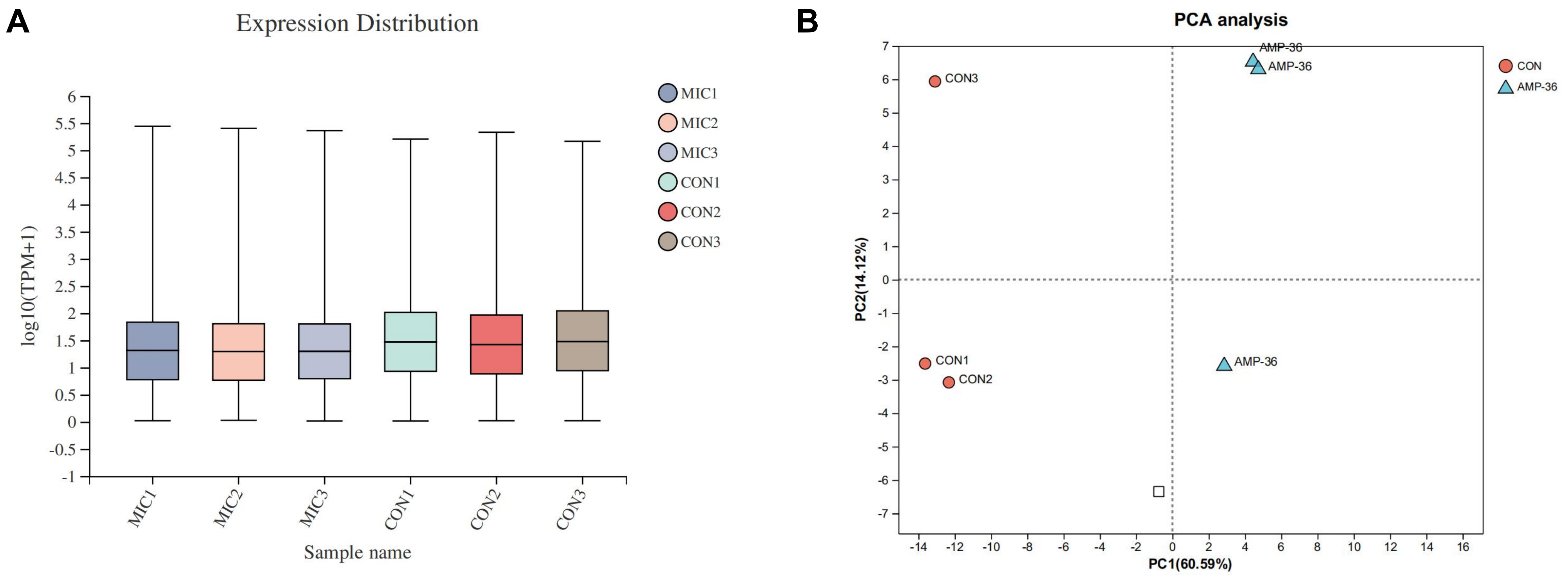

Figure S1. Expression distribution and PCA analysis of samples. (A) Box plot of the expression distribution across samples groups AMP-36 with CON. (B) PCA plot of the expression distribution across samples groups. CON: red circles, AMP-36: blue triangles.

GO enrichment analysis

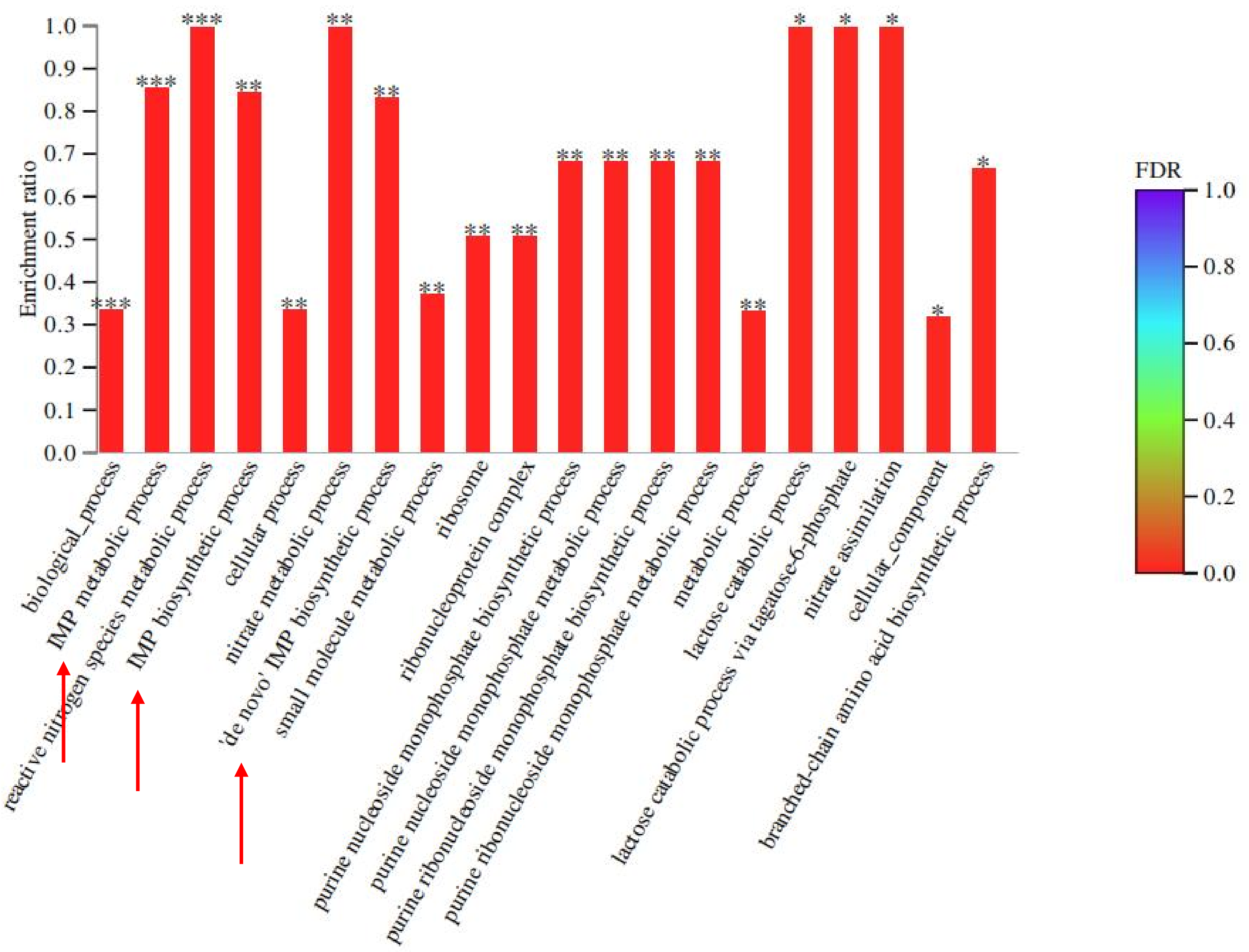

Figure S2. Gene Ontology (GO) enrichment analysis of differentially expressed genes (DEGs) between the AMP-36–treated group and untreated control (CON). Enriched GO terms are shown according to their enrichment ratios, covering biological processes associated with metabolism, biosynthesis, and cellular components. Statistical significance was determined based on adjusted *p* values (****p<0.0001, ***p<0.001, **p<0.01).

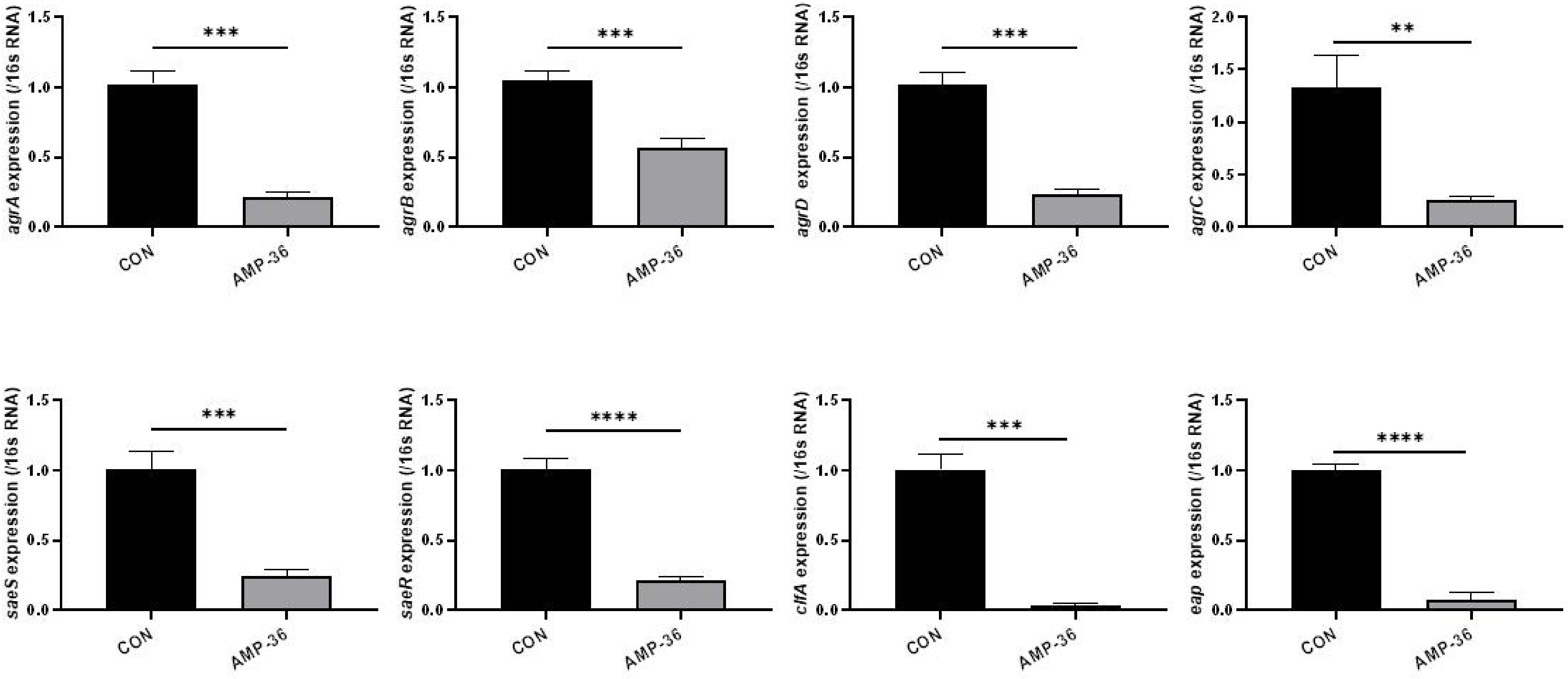

Figure S3. Relative expression of virulence- and regulation-related genes in MRSA following AMP-36 treatment. Gene expression levels were normalized to 16S rRNA and are presented as mean ± SEM. Statistical significance was determined by unpaired Student's t-test ($^{****}p<0.0001$, $^{***}p<0.001$, $^{**}p<0.01$).

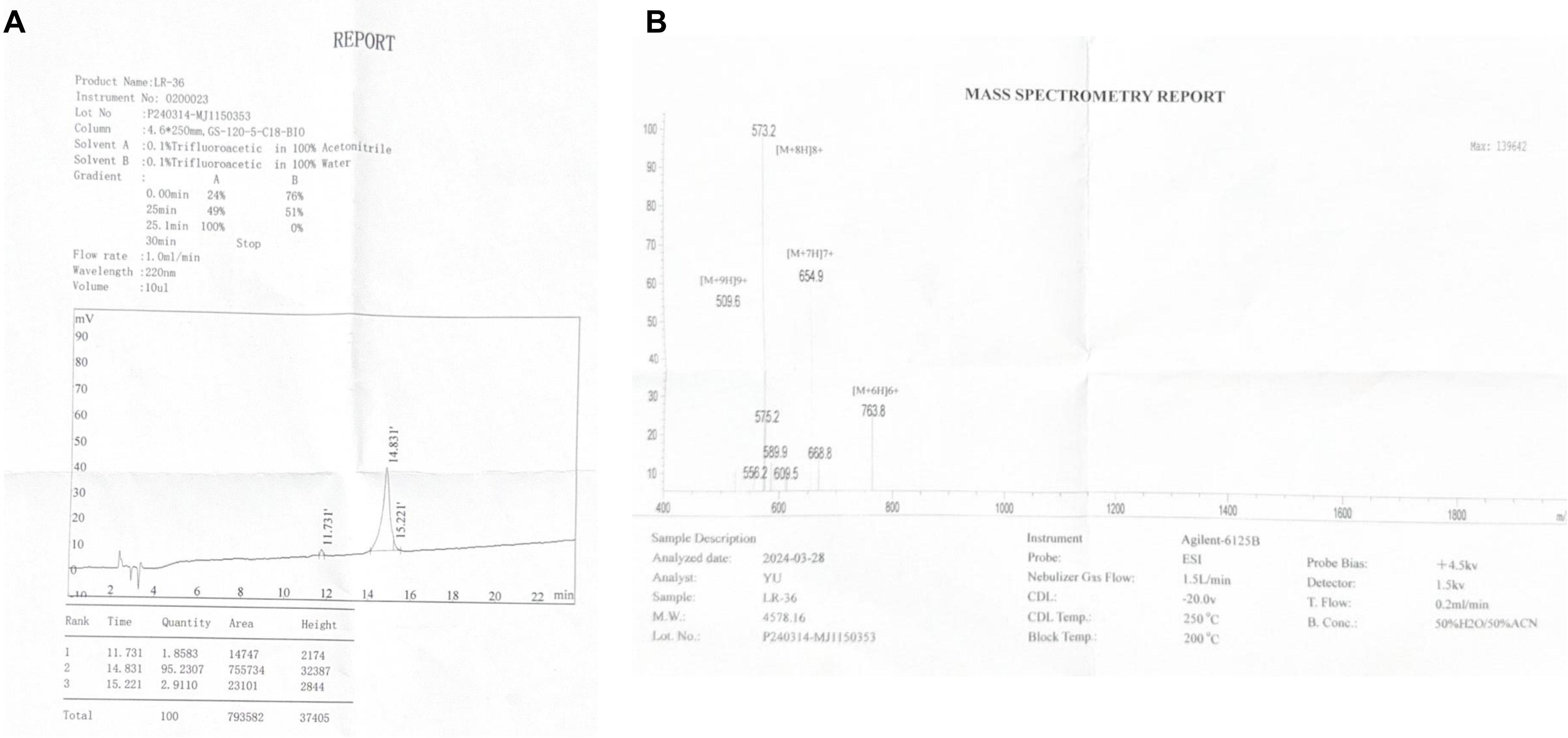

A

REPORT

Product Name:LR-36
Instrument No: 0200023
Lot No :P240314-MJ1150353
Column :4.6*250mm,GS-120-5-C18-BIO
Solvent A :0.1%Trifluoroacetic in 100% Acetonitrile
Solvent B :0.1%Trifluoroacetic in 100% Water

Gradient :	A	B
0.00min	24%	76%
25min	49%	51%
25.1min	100%	0%
30min	Stop	

Flow rate :1.0ml/min
Wavelength :220nm
Volume :10ul

Rank	Time	Quantity	Area	Height
1	11.731	1.8583	14747	2174
2	14.831	95.2307	755734	32387
3	15.221	2.9110	23101	2844
Total		100	793582	37405

B

MASS SPECTROMETRY REPORT

Sample Description

Analyzed date:	2024-03-28	Instrument	Agilent-6125B		
Analyst:	YU	Probe:	ESI	Probe Bias:	+4.5kv
Sample:	LR-36	Nebulizer Gas Flow:	1.5L/min	Detector:	1.5kv
M.W.:	4578.16	CDL:	-20.0v	T. Flow:	0.2ml/min
Lot. No.:	P240314-MJ1150353	CDL Temp.:	250 °C	B. Conc.:	50%H2O/50%ACN
		Block Temp.:	200 °C		

Figure S4. Peptide purity and molecular weight validation of AMP-36. (A) Purity of AMP-36 was evaluated by analytical HPLC with UV detection, demonstrating a purity of >95%. (B) Peptide identity was further confirmed by LC–MS, with the measured molecular mass matching the calculated theoretical mass.
