# Supplementary material for: AMP-36 exhibits potent therapeutic efficacy against MRSA pneumonia through membrane-target mechanism
Source: Sci Rep. 2026 Mar 17;16:13799. doi: 10.1038/s41598-026-44156-6 (PMC13128947; doi:10.1038/s41598-026-44156-6)
Supplement: Supplementary file 2 — Supplementary Information 2. [file 41598_2026_44156_MOESM2_ESM.docx]

Table S1. MIC of of AMP-36 against representative Gram-negative bacteria.

| Strains | MIC |
| --- | --- |
| *P. aeruginosa* ATCC | 16 |
| *P. aeruginosa* (clinical isolate) | 8 |
| *E. coli* ATCC | 4 |
| *E. coli* (clinical isolate) | 4 |

^*^MIC: minimum inhibitory concentration (μg/mL). *P. aeruginosa* (*Pseudomonas aeruginosa*), *E. coli* (*Escherichia coli*). ATCC standard strains and clinical isolates were obtained from the Microbiology Laboratory Department of the Second Hospital of Shandong University.
