# Supplementary material for: AMP-36 exhibits potent therapeutic efficacy against MRSA pneumonia through membrane-target mechanism
Source: Sci Rep. 2026 Mar 17;16:13799. doi: 10.1038/s41598-026-44156-6 (PMC13128947; doi:10.1038/s41598-026-44156-6)
Supplement: Supplementary file 3 — Supplementary Information 3. [file 41598_2026_44156_MOESM3_ESM.pptx]

## Slide 1
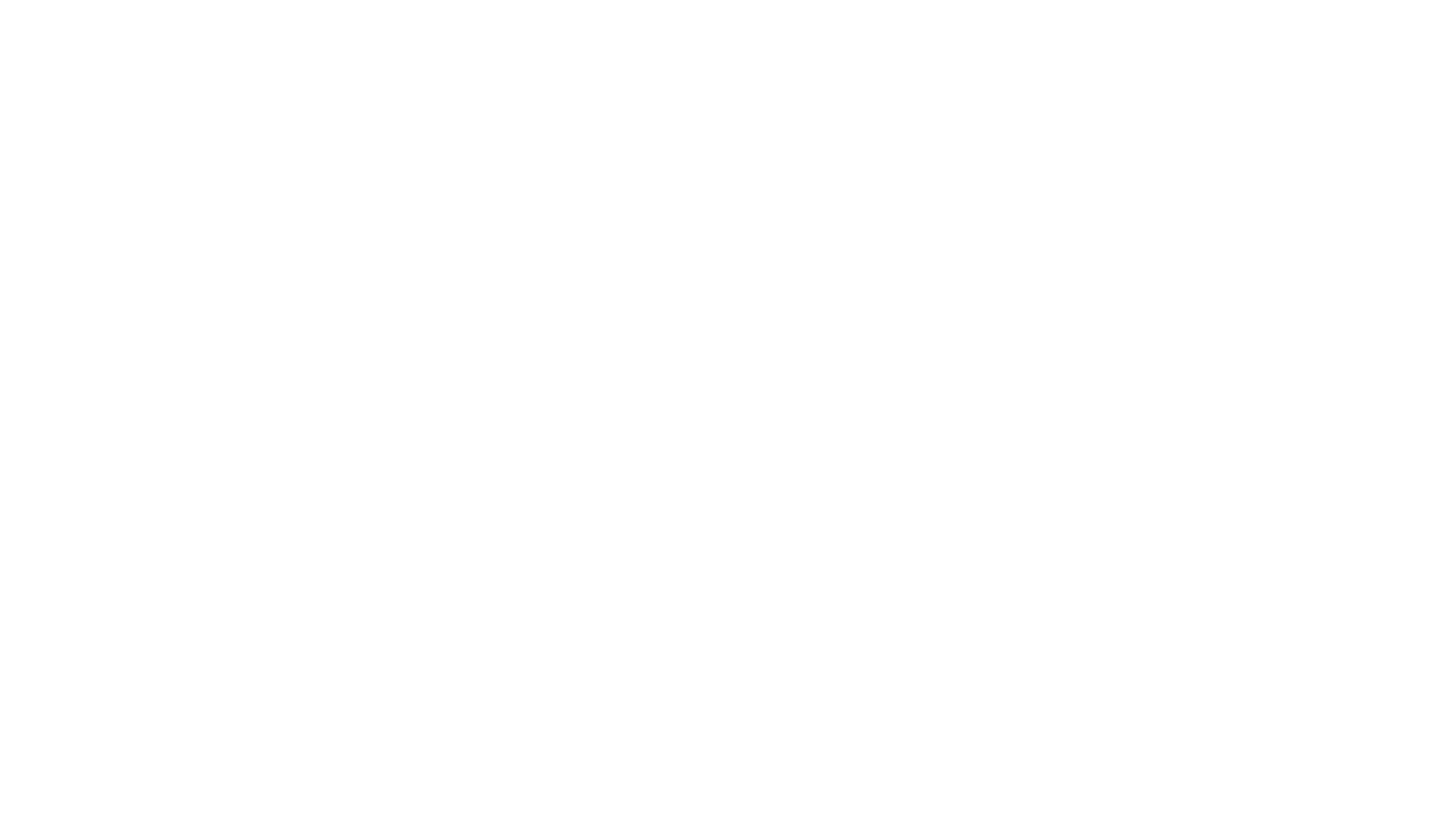

## Slide 2
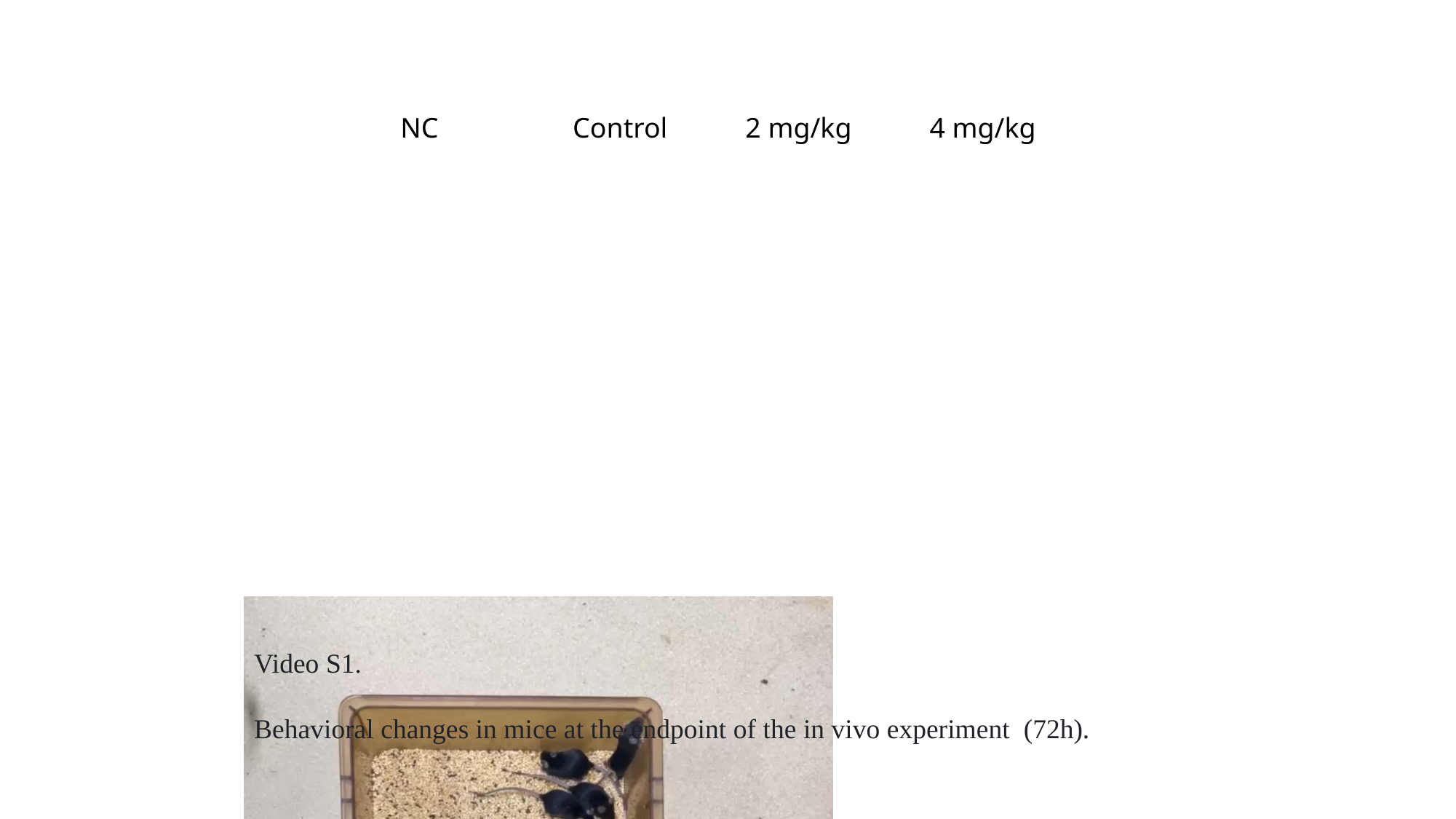

NC Control 2 mg/kg 4 mg/kg
Video S1.
Behavioral changes in mice at the endpoint of the in vivo experiment (72h).
